# Fabrication, micro-structure characteristics and transport properties of co-evaporated thin films of Bi_2_Te_3_ on AlN coated stainless steel foils

**DOI:** 10.1038/s41598-021-83476-7

**Published:** 2021-02-17

**Authors:** Aziz Ahmed, Seungwoo Han

**Affiliations:** 1 Department of Nano-Mechatronics, Korea University of Science and Technology (UST), 217 Gajeong-ro, Yuseong-gu, Daejeon, 305-350 Republic of Korea; 2 Department of Nano-Mechanics, Korea Institute of Machinery and Materials (KIMM), 156 Gajeongbuk-ro, Yuseong-gu, Daejeon, 305-343 Republic of Korea

**Keywords:** Materials science, Materials for energy and catalysis, Thermoelectrics

## Abstract

N-type bismuth telluride (Bi_2_Te_3_) thin films were prepared on an aluminum nitride (AlN)-coated stainless steel foil substrate to obtain optimal thermoelectric performance. The thermal co-evaporation method was adopted so that we could vary the thin film composition, enabling us to investigate the relationship between the film composition, microstructure, crystal preferred orientation and thermoelectric properties. The influence of the substrate temperature was also investigated by synthesizing two sets of thin film samples; in one set the substrate was kept at room temperature (RT) while in the other set the substrate was maintained at a high temperature, of 300 °C, during deposition. The samples deposited at RT were amorphous in the as-deposited state and therefore were annealed at 280 °C to promote crystallization and phase development. The electrical resistivity and Seebeck coefficient were measured and the results were interpreted. Both the transport properties and crystal structure were observed to be strongly affected by non-stoichiometry and the choice of substrate temperature. We observed columnar microstructures with hexagonal grains and a multi-oriented crystal structure for the thin films deposited at high substrate temperatures, whereas highly (00* l*) textured thin films with columns consisting of in-plane layers were fabricated from the stoichiometric annealed thin film samples originally synthesized at RT. Special emphasis was placed on examining the nature of tellurium (Te) atom based structural defects and their influence on thin film properties. We report maximum power factor (PF) of 1.35 mW/m K^2^ for near-stoichiometric film deposited at high substrate temperature, which was the highest among all studied cases.

## Introduction

Thermoelectric materials based devices that operate over a broad range of temperatures are used as power generators and coolers due to their many appealing features, such as offering a clean power source, silent operation, high reliability and long lifetimes. Active cooling modules using thermoelectric cooling devices are in high demand for stabilizing the temperatures of microelectronic systems, where it is becoming increasingly difficult to deliver adequate heat dissipation from high package density microelectronic devices. The dimensionless number known as the figure of merit, ZT, describes the efficiency of thermoelectric material and is expressed as S^2^T/ρk, where S is the Seebeck coefficient, ρ is the electrical resistivity, T is the temperature and k is the thermal conductivity. The output power and efficiency of a thermoelectric device depends primarily on the properties of the thermoelectric material employed^1–5^. Recently, attempts have been made to improve the thermoelectric efficiency of materials based on approaches such as carrier concentration optimization using impurities^6–8^, band structure modifications^8^, and lattice thermal conductivity reduction^3,9^. ZT values can be significantly improved in low-dimensional materials due to the stronger influence of quantum confinement effects and phonon scattering, which enable us to enhance the Seebeck coefficient and decrease the thermal conductivity, respectively^2–4,10–12^.

Bismuth Telluride (Bi_2_Te_3_) is a commercially available thermoelectric material for low temperature applications and is widely employed in conventional industrial applications. It is a heavily doped narrow-bandgap semiconductor with a rhombohedral crystal structure. The rhombohedral lattice implies a hexagonal layered structure where a unit cell contains three quintuple layer sequence, with each layer following the atom scheme Te(1) – Bi – Te(2) – Bi – Te(1)^5,13,14^. Synthesis and optimization of such bismuth chalcogenide thermoelectric materials in low dimensional form is essential due to their applications to the fabrication of integrated micro-scale thermoelectric generators and active Peltier cooling devices^15,16^. Several technologies for the preparation of Bi_2_Te_3_ films, such as thermal evaporation^17–21^, sputtering^16,22–37^, pulse laser deposition (PLD)^38–40^, electrochemical deposition^41,42^, metal organic chemical vapor deposition (MOCVD)^43^ and molecular beam epitaxy (MBE)^44^, have been investigated extensively. For practical device applications and easier integration with microsystems, high quality epitaxial films with sophisticated microstructure and composition control can be obtained using the expensive MOCVD and MBE deposition techniques. The preparation of reasonably performing thermoelectric films by a conventional synthesis method is, however, essential for commercial mass production.

A thermoelectric device can be broadly categorized as either an in- or cross-plane device^15,16^. The motivation for this study was the efficient application of thermoelectric thin films as the p- and n-type segments of a cross plane flexible micro-TE generator. The flexibility of a TE device can become an important feature when they are required to function on a non-planar surface/device. The use of high thermally conductive metal foil substrates is considered essential for achieving higher power density and better thermal management using thermoelectric thin-film micro-devices, by minimizing the thermal loss across the substrate^45^. A major issue that complicates the measurement of thermoelectric properties of samples prepared on conducting/metallic substrates is that conducting substrates short-circuit the current during the measurement of thermoelectric properties^41,42^. Furthermore, most reports on Bi_2_Te_3_ film deposition concern rigid substrates. Some papers have reported thermoelectric thin film deposition on polyimide substrates, which although flexible, are not thermally conductive sufficiently for high power density applications, nor the homogeneity of thin films microstructure and physical properties^17,31,32^. Furthermore, the use of polyimide substrate limits the film processing temperature as they cannot withstand very high temperatures^17^.

The thermoelectric transport characteristics of Bi_2_Te_3_ material are highly sensitive to small compositional variations, a parameter which is hard to precisely control in vapor deposition processes. Furthermore, elemental Tellurium (Te) have similar lattice spacing with Bi_2_Te_3_ through the following relations, *a*_Te_ ≈ *a*_Bi2Te3_ and *c*_Te_ ≈ 1/5 *c*_Bi2Te3_. This lattice match indicates that both materials may be grown together with certain crystallographic relationship i.e. without unfavorable structural distortion. Thermal evaporation of materials is an appealing commercial deposition method due to its simplicity, easy operational oversight, low fabrication cost, scalability to large areas, high throughput and compatibility with micro-patterning. Therefore, we synthesized n-type Bi_2_Te_3_ films by co-evaporation of the constituent elements to obtain thin films with controllable compositions. As the composition of the thin film varied, we placed special emphasis on establishing its relationship with the transport and structural properties of the samples. This study also aims to identify various types of Te based defects that appears to be present in the samples; their nature and effect on properties were examined as well. A 250 μm-thick stainless steel foil was selected as the substrate due to its high thermal conductivity and stability. The thermoelectric thin films were electrically isolated from the conducting substrate by pre-depositing an aluminum nitride (AlN) thin film on the surface of the substrate.

## Methods

### Substrate preparation

Stainless steel foil (Alfa Aesar, Haverhill, MA, USA) was used as the substrate for thin film deposition. To improve the uniformity and reproducibility of the thin film, the stainless steel foils were polished both mechanically and chemically. First, 20 mm square pieces of stainless steel foil were polished with silicon carbide (SiC) sand paper, followed by polishing using a diamond and silica suspension. After polishing, all of the samples were cleaned using alcohol and water. The thickness of the stainless steel foil was reduced from 500 µm to an average value of about 250 µm after the polishing procedure. Unfortunately, further reduction in the substrate thickness was difficult due to the constraints imposed by the polishing apparatus, which limits the flexibility of the foil.

### Film fabrication

#### Aluminum nitride deposition

The AlN thin films were fabricated by direct current (DC) reactive magnetron sputtering (ATC Orion Sputtering system; AJA International Inc., North Scituate, MA, USA) using an Al (99.999% pure) elemental target with a diameter of 2 inches. All of the substrates were cleaned adequately using isopropanol, acetone, ethanol, and deionized water in an ultrasonic agitator, dried with N_2_, then cleaned with O_2_ plasma for 30 s. We ensured a homogenous film composition and thickness by rotating the substrate holder at 10 rpm. Pre-sputtering using Ar plasma was carried out for 10 min before deposition to remove (any) contamination from the target surfaces. The sputtering chamber was pumped down to a pre-deposition vacuum pressure of 5 × 10^−7^ Torr, and the working pressure was set to 8 mTorr with a mixture of 5 sccm of argon (Ar) and 7 sccm of nitrogen (N_2_) as the sputtering gas. The DC power applied to the Al target was 100 W and the experiments were carried out for 3 h, with the substrate temperature maintained at 300 °C and room temperature (RT) for subsequent thermoelectric thin film deposition experiments conducted at high and low substrate temperatures, respectively. The AlN thin films deposited at RT were further annealed at 300 °C for several hours prior to the thermoelectric thin film deposition. Under these deposition conditions, the sputtered Al atoms reacted with nitrogen to form AlN, which was then deposited onto the substrate.

#### Bismuth telluride (Bi–Te) deposition

The Bi–Te thin films were fabricated by the thermal co-evaporation technique using an effusion cell evaporator system (Alpha Plus Co., Ltd., Gyeongsangbuk-do, Korea). The evaporation tubes containing Bi (99.999% pure) and Te (99.999% pure) elemental sources were placed in separate heater assemblies/effusion cells, each of which was connected to an independent power supply and placed at the bottom of the evaporator chamber. The chamber was pumped down to a high vacuum pressure of 3 × 10^−7^ Torr prior to the thin film deposition. We placed a quartz crystal sensor above each source tube to monitor the evaporation flux from that particular source. The substrates were held above the sources and the substrate holder was rotated to obtain a homogenous film thickness and atomic composition. By adjusting the power applied to the effusion evaporator source, the evaporation rate was controlled and a series of thin films with a variety of compositions was obtained. In the series of experiments where the substrates were maintained at a temperature of 300 °C during deposition, the Bi deposition rate was fixed at 1.3 Å/s, whereas the deposition rate for Te was varied over 3.5–4.5 Å/s. These samples were further annealed at 220 °C for 3 h inside the vacuum chamber. The Bi rate was fixed at 1.8 Å/s and the Te rate was varied between 1.9–2.8 Å/s in the case of the experiments conducted with substrates maintained at RT during deposition. These samples were annealed at 280 °C for 3 h inside the vacuum evaporator chamber.

### Film characterization

#### Crystal structure, thickness, and composition

The microstructure, composition, crystal structure and orientation of the thin films were evaluated by high resolution field emission scanning electron microscopy (FE-SEM), energy dispersive X-ray spectroscopy (EDS) and X-ray diffraction (XRD), respectively. The details of the characterization methods have been published elsewhere^7,12^.

#### Electrical and thermoelectric properties

The in-plane electrical and thermoelectric properties were determined using a commercially available TFTEP-800 (SEEPEL Co. Ltd., Gunpo, Korea) measurement system. The electrical resistivity of the thin films was measured using the conventional four point probe method. The Seebeck coefficient was measured by thermally connecting the two opposite sides of the sample to two different heaters at different temperatures, thus creating a temperature difference (ΔT). The resulting potential difference (ΔV) was measured and the Seebeck coefficient was calculated as the ΔV/ΔT ratio. The power factor (PF), defined S^2^/ρ, was calculated using the experimental data for the Seebeck coefficient and the electrical resistivity. The measurement errors for the calculated PF were maintained at < 10% for all cases reported.

## Results and discussion

**Figure 1:**
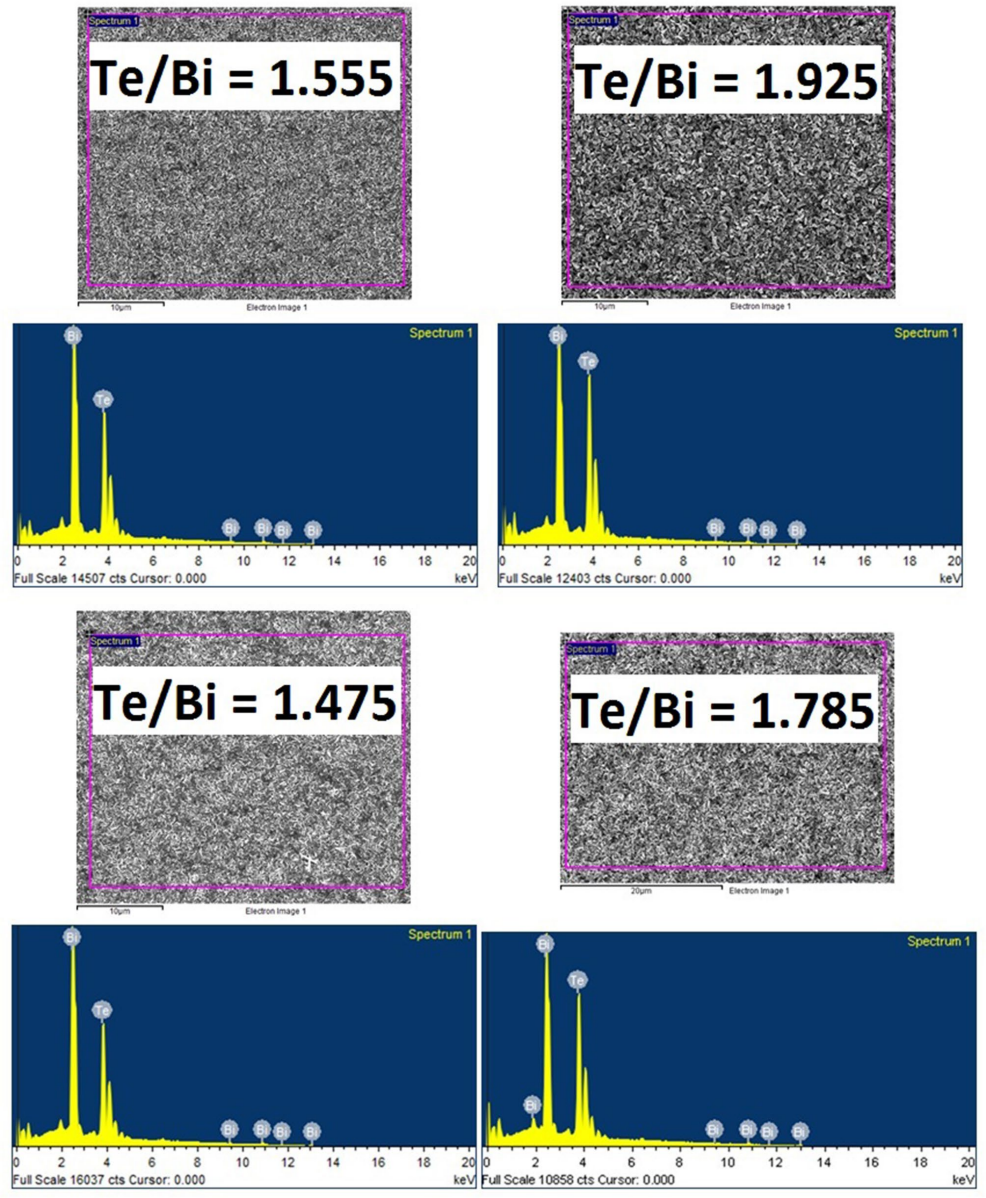
EDS mapping images and spectrums of the selected bismuth telluride (Bi–Te) thin films deposited at high substrate temperatures.

**Figure 2:**
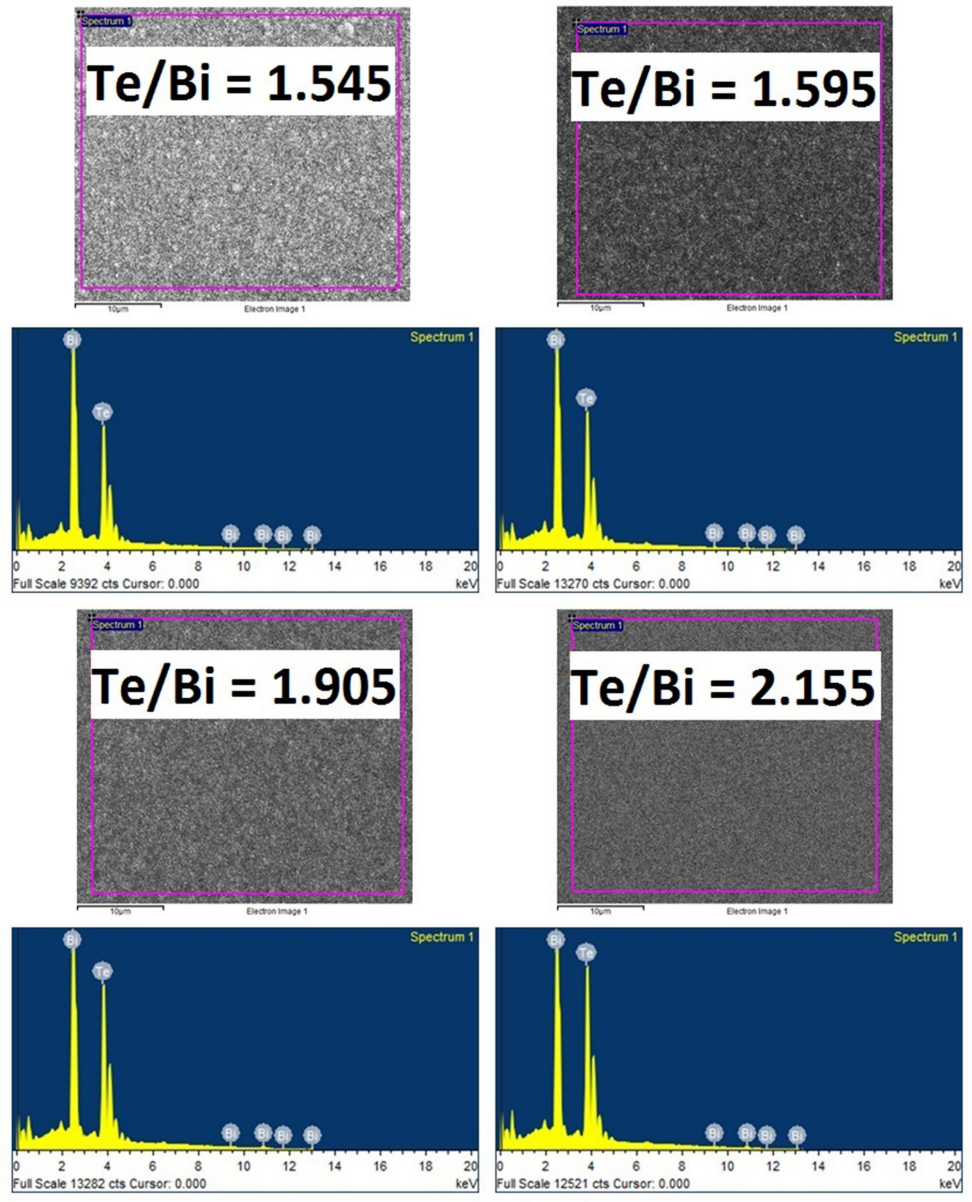
EDS mapping images and spectrums of the selected Bi–Te thin films after thermal annealing.

A Bi–Te-based thin film samples series differing in compositions were deposited at different substrate temperatures and their thermoelectric properties were studied. AlN was selected as the electrically insulating material^46^ due to its reported good thermal conductivity^47^. This is beneficial for applications in a vertical type thermoelectric device, which requires efficient heat transfer from the heat source to the thin film thermocouple through the substrate assembly. However, in this paper, we focused exclusively on the electrical properties of the insulation layer. Furthermore, as mentioned previously, the thickness of the substrate foil was approximately 250 µm. While the thickness of the foil limits its flexibility, it ensures good thermal contact of the substrate with the heater, thus avoiding large temperature gradients across the sample during the film deposition or annealing processes. This ensures uniformity in the microstructure and thermoelectric properties throughout the sample surface. The Te/Bi ratio (and therefore the composition) was varied by fixing the Bi evaporation rate and varying the Te evaporation rate. The film composition was measured several times at different locations on the samples, but only the averaged values are reported here. Tables 1, 2 and 3 shows the compositions and Te/Bi ratio of the prepared thin films in their final state after their respective annealing step. For ease of reference, all of the thin films are named after their identified Te/Bi composition ratio. A Te/Bi ratio of 1.5 corresponds to stoichiometric Bi_2_Te_3_ composition. The typical EDS mapping images and spectrums for the two prepared sets of thin films are presented in Figs. 1 and 2. The EDS results clearly illustrates that the prepared thin films possess varying concentrations of Te atoms which enables us to investigate the effect of Te based defects on the Bi_2_Te_3_ thin film properties. The measured film thicknesses ranged between 1 and 2 μm and therefore were thick enough to reduce their internal resistance.

**Table 1:** The compositions and Te/Bi ratio of the Bi–Te thin films fabricated with a pre-deposited Cr layer at high substrate temperature.

Nominal composition	Nominal Te/Bi	Actual composition	Actual Te/Bi
Bi_2_Te_3+x_/x = 0.1	1.55	Bi_2_Te_3.11_	1.555
Bi_2_Te_3+x_/x = 0.2	1.60	Bi_2_Te_3.19_	1.595
Bi_2_Te_3+x_/x = 0.4	1.70	Bi_2_Te_3.46_	1.730
Bi_2_Te_3+x_/x = 0.8	1.90	Bi_2_Te_3.85_	1.925

**Table 2:** The compositions and Te/Bi ratio of the Bi–Te thin films fabricated without a pre-deposited Cr layer at high substrate temperature.

Nominal composition	Nominal Te/Bi	Actual composition	Actual Te/Bi
Bi_2_Te_3+x_/x = −0.1	1.45	Bi_2_Te_2.93_	1.465
Bi_2_Te_3+x_/x = 0.0	1.50	Bi_2_Te_2.95_	1.475
Bi_2_Te_3+x_/x = 0.0	1.50	Bi_2_Te_2.99_	1.495
Bi_2_Te_3+x_/x = 0.1	1.55	Bi_2_Te_3.06_	1.530
Bi_2_Te_3+x_/x = 0.6	1.80	Bi_2_Te_3.57_	1.785

**Table 3:** The compositions and Te/Bi ratio of the annealed Bi–Te thin films.

Nominal composition	Nominal Te/Bi	Actual composition	Actual Te/Bi
Bi_2_Te_3+x_/x = 0.0	1.50	Bi_2_Te_2.99_	1.495
Bi_2_Te_3+x_/x = 0.1	1.55	Bi_2_Te_3.09_	1.545
Bi_2_Te_3+x_/x = 0.2	1.60	Bi_2_Te_3.19_	1.595
Bi_2_Te_3+x_/x = 0.8	1.90	Bi_2_Te_3.81_	1.905
Bi_2_Te_3+x_/x = 1.3	2.15	Bi_2_Te_4.31_	2.155

### Structural and thermelectric properties of thin films synthesized at high substrate temperatures

We carried out our experiments while maintaining the temperature of the substrate at 300 °C. Further annealing at 220 °C for 3 h under a high vacuum was carried out to reduce thermal stress and defects in the thin films. The lower annealing temperature of 220 °C ensured that any Te loss through sublimation is completely prevented. The thin film samples discussed in this section were characterized after this annealing step. The substrate temperature of 300 °C was considered sufficient to obtain phase pure Bi_2_Te_3_ and prevent the formation of other Bi–Te-based phases. The high substrate temperatures caused sublimation of Te from the deposited thin film, which compelled us to use a high Te evaporation rate when preparing excess Te samples. A thin layer of chromium (Cr) metal can help such samples to withstand thermal shocks and increase the adhesion of the thin film to the substrate or any other underlying layer^48^. Therefore, some of the thermoelectric thin films were fabricated on a 20 nm pre-deposited thin layer of Cr and the transport properties were compared to those without an adhesion layer.

**Figure 3:**
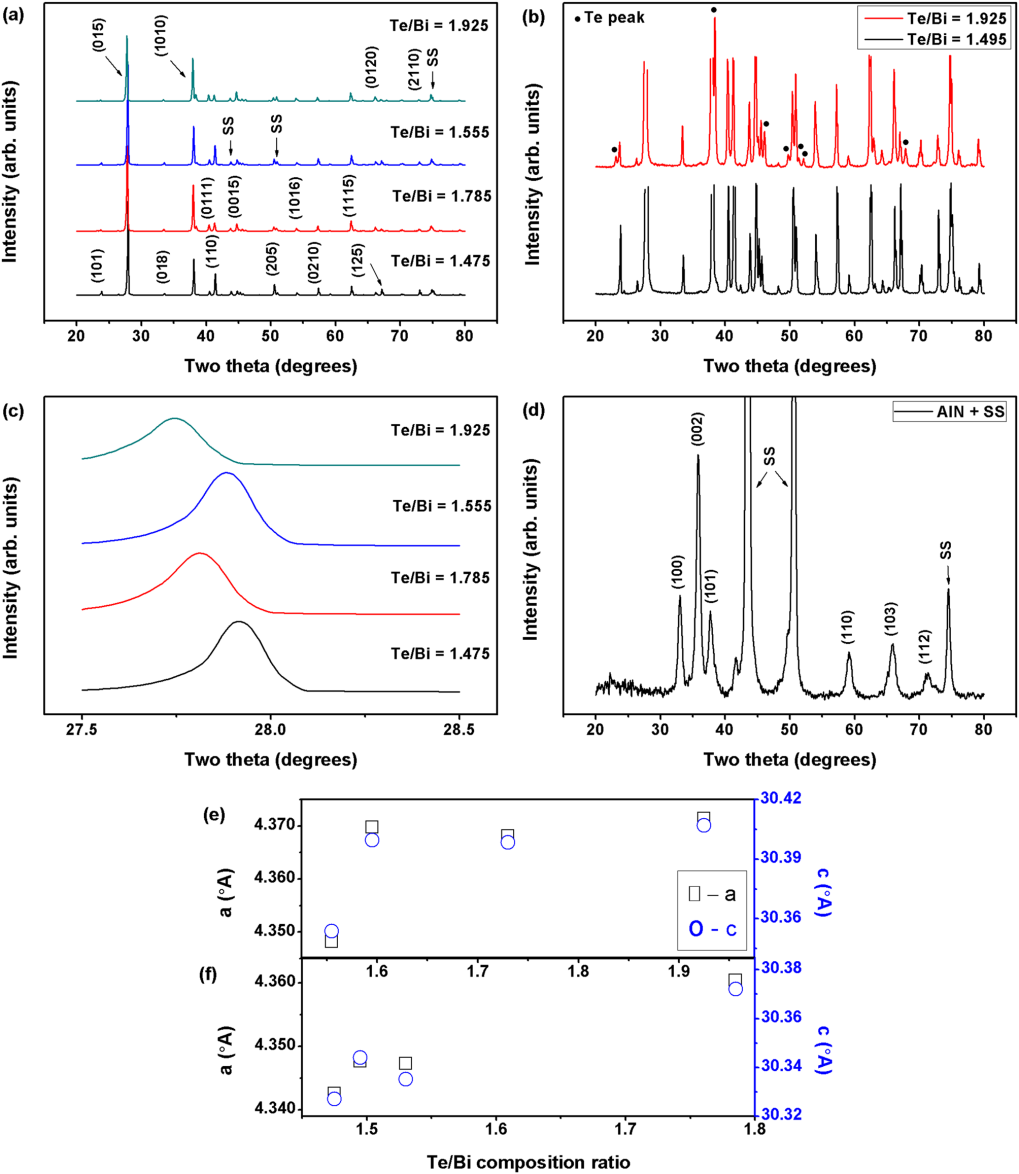
Room temperature (RT) X-ray diffraction (XRD) patterns for the (**a**) selected bismuth telluride (Bi–Te) thin films deposited at high substrate temperatures, (**b**) selected thin films where high intensity Bi–Te peak data was removed for better visualization of the less intense Te peaks, which were observed due to the deviation from stoichiometry, (**c**) selected thin films illustrating the peak shift associated with the Bi–Te main (015) peak, and (**d**) aluminum nitride (AlN) thin film deposited on polished stainless steel foil at high substrate temperatures. The a-axis lattice parameter and c-axis lattice parameter of the Bi–Te thin films as a function of Te/Bi composition ratio, determined from XRD patterns: (**e**) with adhesion layer and (**f**) without adhesion layer.

Figure 3a shows XRD patterns of the selected Bi–Te thin films with and without the Cr adhesion layer. The XRD peaks suggest that all of the samples were polycrystalline with well-developed phases. The presence of several different diffraction peaks indicates multiple grain orientations or existence of a secondary phase. The experimental data were evaluated in terms of the standard powder XRD pattern for polycrystalline Bi_2_Te_3_, and the obtained peak positions were found to agree well with the standard patterns (PDF#00-015-0863). In this set of experiments, diffraction peaks of notable intensities could not be spotted at diffraction angles < 20° which was consistent with the standard powder patterns. The Two-Theta range selected in Fig. 3 is therefore representative of the clearly distinguishable and high intensity Bi_2_Te_3_, Te and substrate peaks obtained at diffraction angles ≥ 20°. A typical multi-oriented rhombohedral Bi_2_Te_3_ crystal structure was observed where the out-of-plane diffraction peak corresponding to the (015) lattice plane was more intense than the (1010) and (110) lattice plane peaks. There was little difference in the Bi_2_Te_3_ XRD peaks among the samples, indicating that varying the composition had little effect on the predominant orientation (and therefore the crystal structure) of the films. Upon closer inspection, however, some additional low intensity peaks were observed for samples that were relatively off-stoichiometric. These additional peaks are shown in Fig. 3b, where the higher intensity Bi_2_Te_3_ peaks were removed for better visualization. These peaks were identified and matched with those of Te metal, which was present in crystallized metallic form as a secondary phase. Overall, we only observed Bi_2_Te_3_ or Te phases in the XRD patterns, which confirms the phase purities of the thin film samples. Furthermore, an increase in the Te/Bi ratio resulted in a peak shift towards lower diffraction angles as illustrated for the main (015) peak in Fig. 3c. Figure 3d shows the XRD patterns of an AlN thin film, which we used as an electrical insulation layer in these experiments. The AIN was deposited onto a polished stainless steel foil at high temperatures. These AlN peaks could not be clearly observed for XRD experiments performed after the deposition of thermoelectric thin films. We have determined the *a*-axis and *c*-axis lattice parameter from the XRD data; for the most stoichiometric sample (Te/Bi = 1.495), they were found to be 4.35 Å and 30.34 Å respectively. These values were slightly smaller than the bulk lattice constants *a* = 4.38 Å and *c* = 30.45 Å^49^. Both lattice parameters increases with increasing Te/Bi composition ratio (Fig. 3e, f) which indicates composition induced structure evolution in the thin films. This observation was consistent with the (015) peak shift towards lower diffraction angles that was observed for excess Te thin films. The three types of defects likely to exist in off-stoichiometric Bi_2_Te_3_ are antisite, vacancy and interstitial defects. Vacancies may be ignored in this study due to their higher formation energy (about 1 eV^50^) than the antisite defects (0.4 eV^51^). Antisite defects are usually considered as the main type of structural defects in Bi_2_Te_3_ material in case of deviation from stoichiometric compositions^49^. The observed increase in the lattice parameters with Te/Bi ratio in this study however indicates that some of the excess Te atoms may have acted as interstitials in the Bi_2_Te_3_ unit cell.

**Figure 4:**
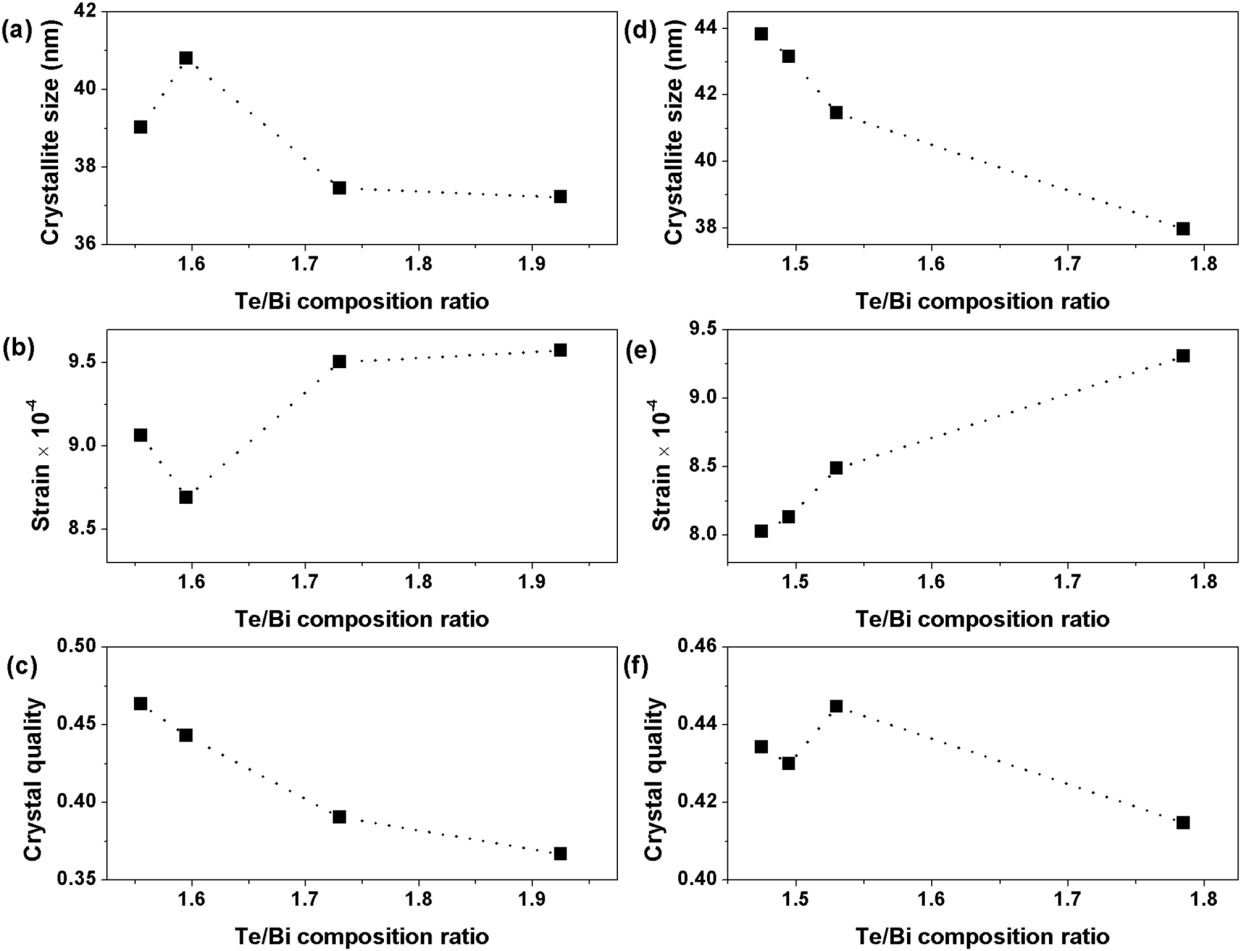
The average crystallite size, lattice strain and crystal orientation/quality of the Bi–Te thin films fabricated at high substrate temperatures.

Figure 4 shows the compositional dependence of the crystallographic properties of the Bi–Te thin films such as average crystallite size, micro-strain and crystal quality/orientation. The average nano-crystallite size, D, of the thin films (Fig. 4a, d) was estimated from the XRD peaks data using Scherrer’s equation^52–54^ given by the relation kλ/βcosθ, where k is a dimensionless shape factor/constant with a value 0.9, λ is the wavelength of the Cu-Kα X-ray radiation (0.154056 nm), β is the full width at half maximum (FWHM), and θ is the Bragg angle. The crystallite size of all the thin films ranged between 37 and 44 nm values and it slightly decreases when the Te/Bi ratio of the thin films was raised. It should be noted here that the nano-crystallite size calculated through the Scherrer’s equation is assumed to be the coherently diffracting domain size and, in most cases, is not the same as the grain size. In this study, grains are understood to be the agglomerations of many crystallites. The internal micro-strain, ε, is closely related to the concentration of lattice imperfections in the thin films. The micro-strain of the films was estimated using the relation βcosθ/4^53–55^. As shown in Fig. 4b, e, in general, the strain in the thin films tended to increase with the Te/Bi composition ratio. These results provide evidence that some of the excess Te atoms may have become interstitials inside the Bi_2_Te_3_ unit cells instead of being present at the crystal boundaries. These interstitials are then also responsible for the observed lattice expansion in the higher Te content thin films. Figure 4c, f shows the crystal orientation/quality of the films which was determined from the intensity ratio of the most intense (015) peak to the sum of intensities of all peaks, Σ[hkl]. The highest intensity ratio among all studied cases, (015)/Σ(hkl) ~ 0.45, was calculated for near stoichiometric thin films and it decreases with an increase in the thin film Te content. These results indicate that the thin films deposited at high substrate temperatures do not have any dominant crystal orientation and could therefore be labelled as randomly oriented. Therefore, the introduction of Te based defects in Bi–Te thin films results in a slightly smaller nano-crystallite size, higher strain and lower film crystallinity.

**Figure 5:**
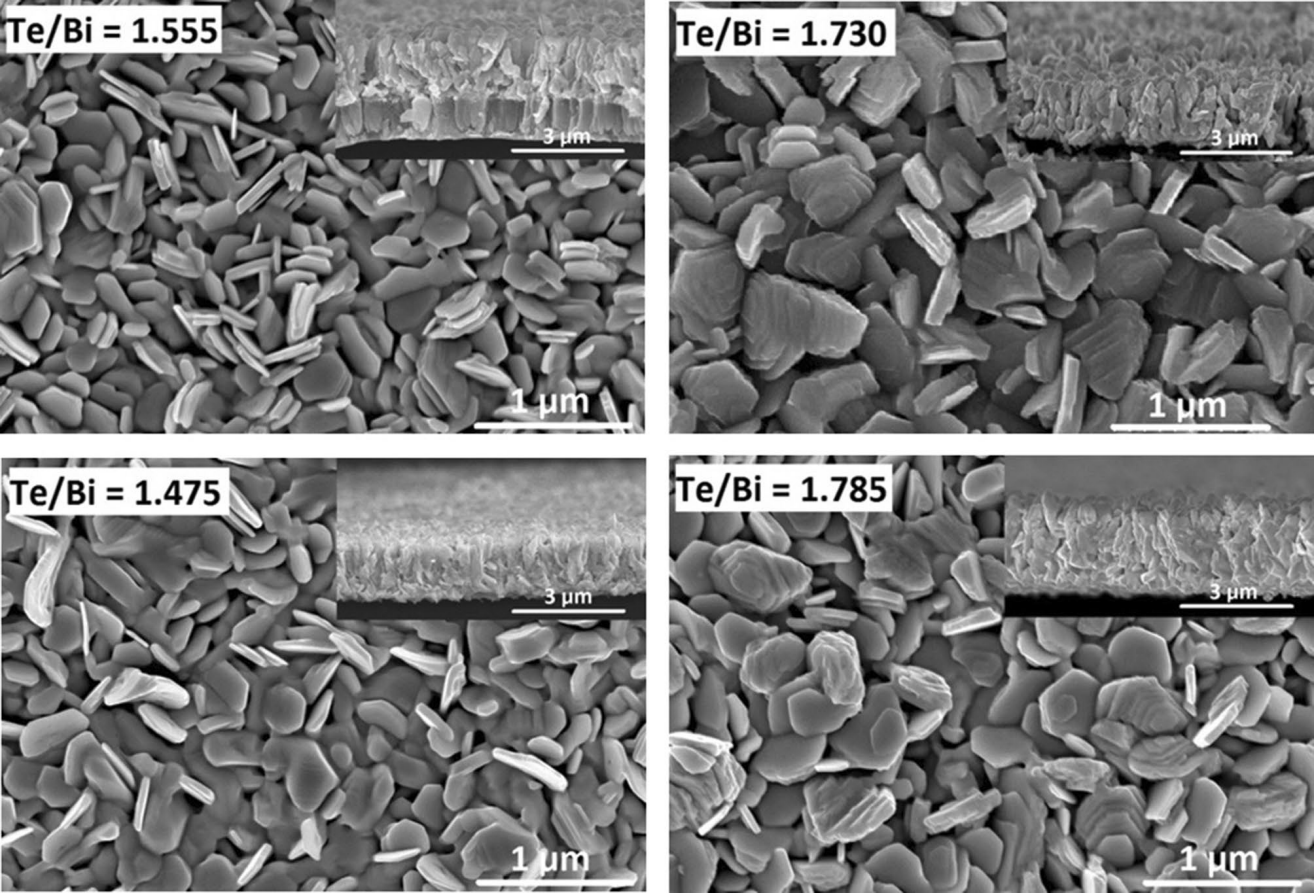
Scanning electron microscopy (SEM) images showing the surface and cross-sectional (inset) morphologies of the selected Bi–Te thin films fabricated at high substrate temperatures: top row with adhesion layer and bottom row without adhesion layer.

The SEM images in Fig. 5 show the surface and cross-sectional morphologies of the thin film samples, revealing large and multi-oriented grains closely embedded in the film surface, which further confirms the polycrystalline structure of the deposited thin films. The observed hexagonal grains were consistent with the rhombohedral crystal structure inferred from the XRD analysis. Due to the high substrate temperature, more thermal energy was available during the film growth, causing large grains to form quickly by the coalescence or growth of smaller ones. The high available kinetic energy and deposition rate were also responsible for the observed multi-oriented structure, as the deposited atoms aggregated quickly and therefore did not have enough time to reach the proper lattice points for ordered single oriented growth. Overall, the thin films seem to have followed the island growth model and the grain structure of the thin films shows an obvious effect of composition on film morphology. The cross-sectional images of the samples reveal fast film nucleation and growth in the vertical direction in comparison to the in-plane direction, which causes the grains to form a well-defined out of plane columnar microstructure. Thin film detachment from the foil shown in the cross-sectional images occurred when preparing the samples for SEM analysis. AlN layer is shown to possess a polycrystalline columnar microstructure. The adhesion of the thermoelectric thin film to the AlN layer was visibly improved by the introduction of the Cr adhesion layer, because no delamination occurred between the two thin films when making a cross-cut sample for SEM.

**Figure 6:**
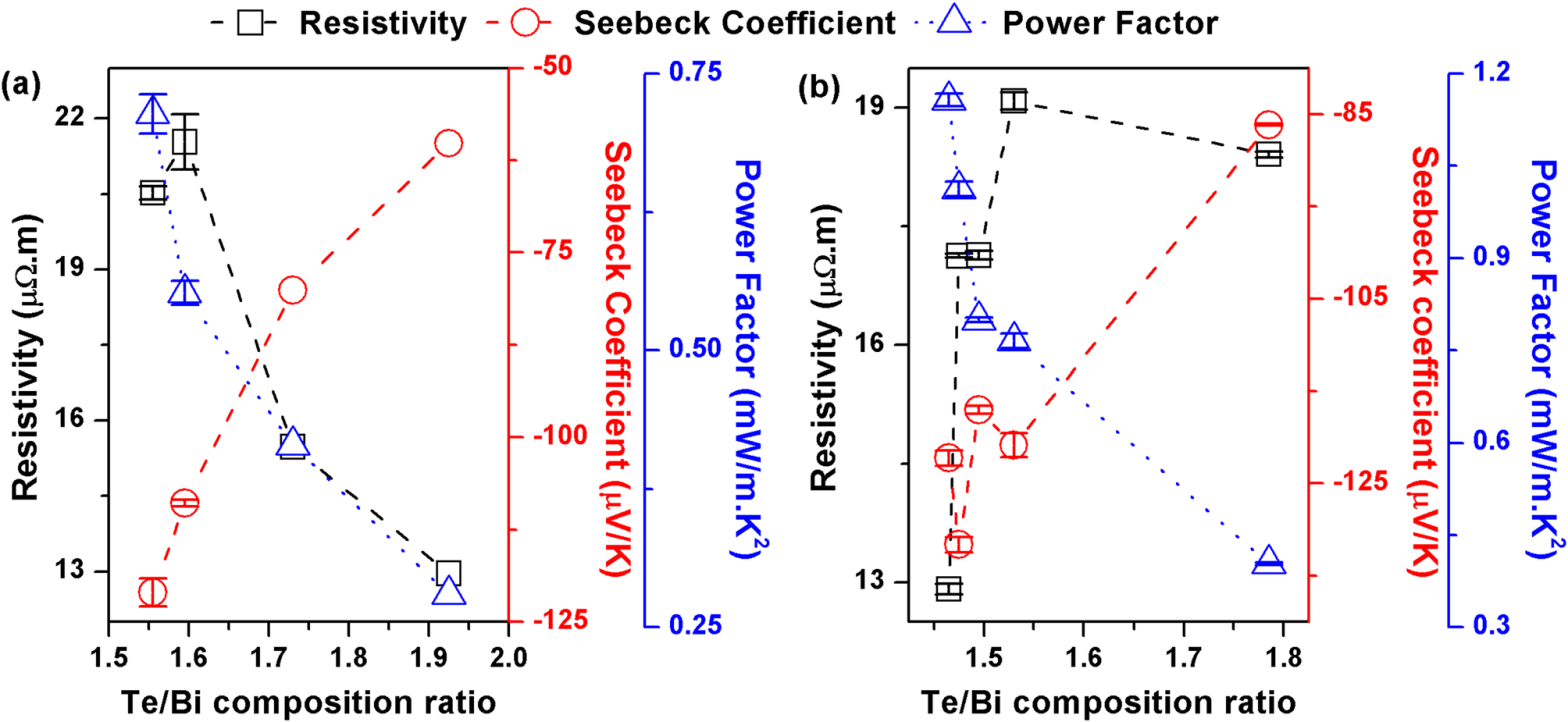
The electrical resistivity, Seebeck coefficient and power factor (PF) of the Bi–Te thin films prepared at high substrate temperatures: (**a**) with adhesion layer and (**b**) without adhesion layer.

The electrical resistivity of the samples was measured and the results are shown in Fig. 6a, b for thin films with and without the Cr adhesion layer, respectively. An increase in the Te/Bi ratio of the thin film beyond stoichiometry resulted in a significant decrease in electrical resistivity of samples prepared with an adhesion layer. It has been reported for Bi_2_Te_3_ thin films that the formation energy for an antisite defect is low and electrons are generated by Te_Bi_ antisite defects in Bi_2_Te_3_, which are formed by the occupation of Bi sites by excess Te atoms^49^. The lower resistivity of the off-stoichiometric samples in Fig. 6a could thus be explained by considering the high concentration of antisite donor defects. The slight increase in the electrical resistivity with increasing Te/Bi ratio for samples prepared without Cr layer is a result of carrier scattering due to excess Te atoms. In off-stoichiometric samples, the interface between Te atom based defects and Bi_2_Te_3_ matrix can create a localized strain field which act as a potential barrier affecting carrier transport. This behavior was not clearly observed in samples prepared with the adhesion layer probably because of the relatively higher background carrier density provided by the Cr atoms which masked any effect of carrier scattering. It therefore appears that excess Te in thin films have both an electron donor effect and a current blocking effect and these effects compete together to influence the electrical properties of the films. Due to their high electrical resistivity and a different thermal conductivity, crystallized Te atoms influences both the electron and heat transport in the thin films. The existence of the excess metal atom based defects between the cleavage planes in off-stoichiometric thin films may increase the electrical resistivity if they are assumed to be oriented normal to the electron flow direction. The above observation was further confirmed by hall measurements conducted on few mm side square samples with Te/Bi ratio of 1.495 and 1.785. The hall mobility decreased from 3.75 × 10^−3^ to 2.25 × 10^−3^ m^2^/V s whereas the electron concentration increased from 8.9 × 10^25^ to 13.6 × 10^25^ m^-3^ as the Te/Bi ratio in the thin film was raised. The hall measurements are however discussed sparingly in this study due to certain experimental difficulties. The electrical resistivity results suggest that the deposition temperatures were sufficient to produce high enough crystallization while simultaneously preventing excessive Te re-evaporation.

The Seebeck coefficient (shown in Fig. 6a, b for thin films with and without a Cr layer, respectively) indicates strong dependence on the thin film composition. The Seebeck coefficient was larger for the samples with compositions closer to phase stoichiometry. The highest Seebeck coefficient was measured for thin film with slight Te deficiency (Te/Bi = 1.475 sample) prepared with no adhesion layer. The Seebeck coefficient tended to decrease as the Te/Bi ratio of the thin film increased. This was due to the higher n-type defect concentration in the off-stoichiometric samples, which increased the Fermi level and reduced the thermopower. This decrease of the Seebeck coefficient in high Te/Bi ratio films also suggest that all types of Te atom based structural defects, regardless of which one is dominant, acted as n-type dopant in the Bi_2_Te_3_ thin films. The Seebeck coefficient of the thin films was negative, which confirms application as an n-type thermoelectric material. According to the previous reports on bulk material, Bi_2_Te_3_ with Te/Bi ratio < 1.5 should be p-type^56^. However, our results have found Te deficient Bi_2_Te_3_ thin films to be n-type at least for composition ranges closer to phase stoichiometry. This may indicate the presence of an alternate doping mechanism in Bi_2_Te_3_ thin films which is related to structural defects introduced during the thin film preparation process.

The PFs of the thin films were calculated by dividing the square of the Seebeck coefficient by the electrical resistivity (Fig. 6a, b). Larger PFs were observed in the near stoichiometric thin films with larger Seebeck coefficients. Due to having a large Seebeck coefficient and a low electrical resistivity, the thin film with a slight Te deficiency (Te/Bi = 1.465 sample) exhibited the highest PF, of approximately 1.13 mW/m K^2^. Thus, we can conclude that a deviation from stoichiometry results in significant changes in the electronic band structure, as well as to repositioning of the Fermi level, which drastically affects the thin film transport properties.

We observed the largest PF, of 1.13 mW/m K^2^, in a thin film deposited without a Cr adhesion layer. This was larger than the maximum PF of about 0.7 mW/m K^2^ obtained for the thin films deposited with the Cr adhesion layer. The thermoelectric properties of the thin films were sensitive to even slight differences in composition, and it was difficult to prepare thin films with exactly the same composition with and without adhesion layers. The question therefore remains as to whether the 20 nm thick Cr adhesion layer had a significant effect on the transport properties of the Bi_2_Te_3_ thin film under the prevailing experimental conditions. A more extensive investigation and more experimental data are needed.

### Structural and thermoelectric propoertes of thin films synthesized at low subsrtare temperatures then annealed

These experiments were carried out whilst maintaining the substrate at RT. This is beneficial because thin films deposited at lower temperatures are more suitable for use in device fabrication due to having a low surface roughness. It is also more convenient to grow thicker thin films at low deposition temperatures. The as-deposited thin films were found to be amorphous. Post-annealing was performed at 280 °C under a high vacuum, to increase the degree of crystallinity and reduce the defects, such as the grain boundaries, atomic scale point defects and vacancies that are known to occur in the amorphous state of Bi–Te and enhance the phase purity of the thin films. Te is reported to have a high diffusivity/vapor pressure at 300 °C and excess Te atoms can evacuate either through grain boundary diffusion passage from the interior to the film surface or simply evaporate^17,57,58^. The annealing was therefore performed in a controlled environment where the annealing temperature was selected to prevent the re-evaporation of volatile Te from the thin film and therefore preserve the composition achieved in the as-deposited state. This enabled us to develop guidelines where as-deposited film compositions obtained for a given Bi and Te evaporation flux could be related to a specific (after annealed) microstructure and preferred crystal orientation. However, this self-imposed temperature constraint does compromise the achievable thermoelectric efficiency. The compositions mentioned in this section were re-measured values obtained after thermal annealing of the thin films.

**Figure 7:**
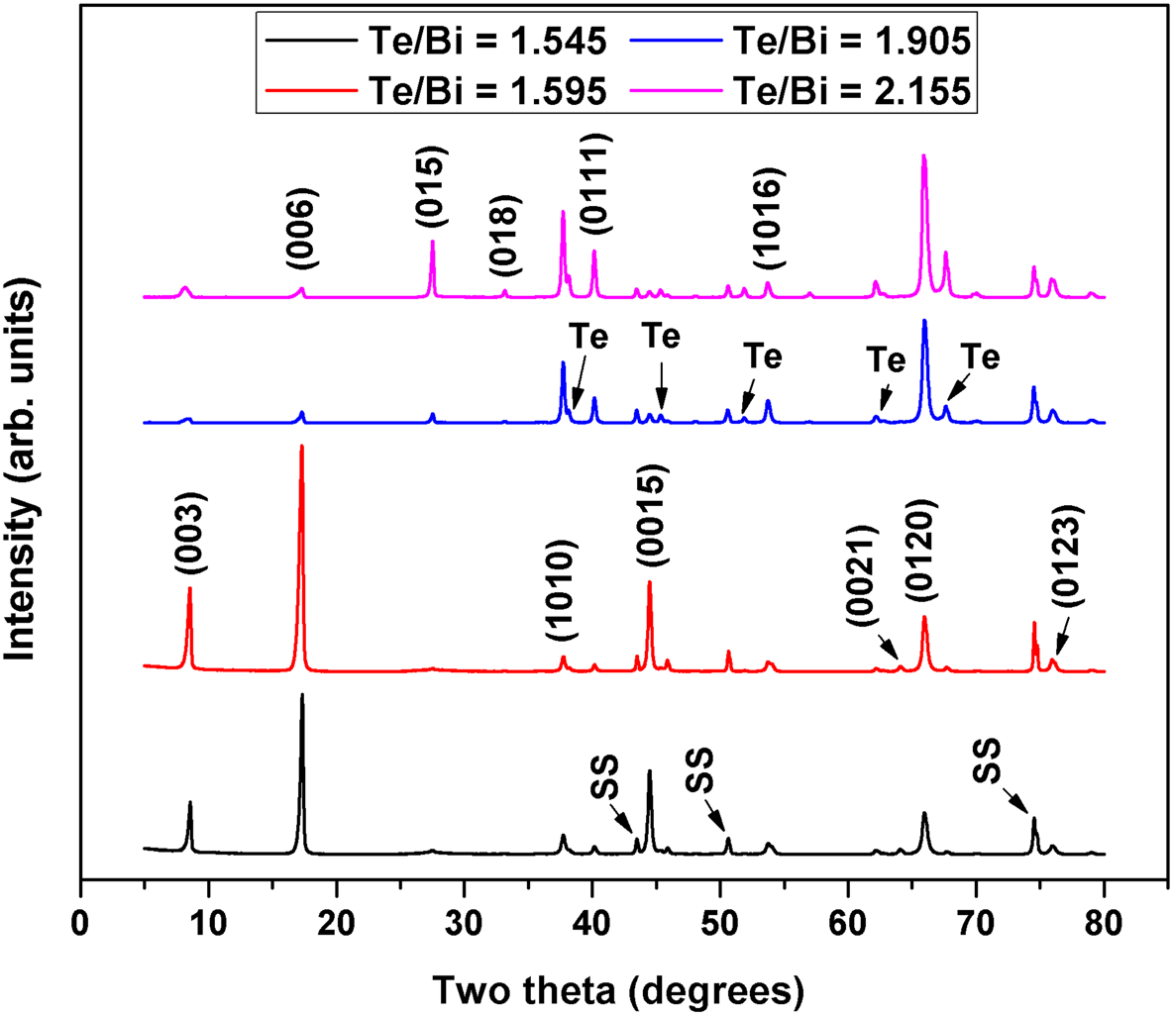
RT XRD patterns for the Bi–Te thin films after thermal annealing.

Figure 7 shows XRD patterns of the Bi–Te thin films after thermal annealing. The impact of deposition temperature (and the heat treatment process that follows) on the thin film nucleation process and growth orientation is well pronounced from the diffraction patterns. In the cases of samples with compositions closer to stoichiometry, we observed strong diffraction peaks corresponding to the (003), (006) and (0015) basal planes. This indicates that the film growth occurred along the natural growth orientation (i.e., along the c-axis of the Bi_2_Te_3_ crystal) and the c-axis of the film was aligned normally to the substrate plane. The existence of several (00* l*) peaks, among them the (006) peak, which was the strongest, may indicate some degree of poly-crystallinity in the annealed thin film samples. This is unlike the (015)-peak based multi-oriented crystal structure observed in the samples deposited at higher substrate temperatures. These results indicate that highly textured, if not epitaxial, thin film growth with a classical layered microstructure was possible for annealed Bi_2_Te_3_ films with near stoichiometric compositions. On the other hand, there were considerable differences in crystal orientation and individual peak intensity between the highly off-stoichiometric excess Te samples and the stoichiometric samples. In the case of off-stoichiometric samples, the (015), (1010) and (0120) plane diffraction peaks were among the most intense peaks. We also observed additional peaks representing crystallized excess Te that did not form a compound with the Bi atoms. These differences in crystal structure and (as discussed later) morphology are due to gradual increase in Te based defects in film structure and the resulting lattice stress and grain reorientation. At a certain defect level, change in film orientation becomes thermodynamically favorable. The XRD patterns suggest that the annealing conditions were sufficient to promote crystallization in the prepared samples, and that the c-axis was the dominant orientation for the near stoichiometric thin films under the prevailing deposition and heat treatment conditions.

**Figure 8:**
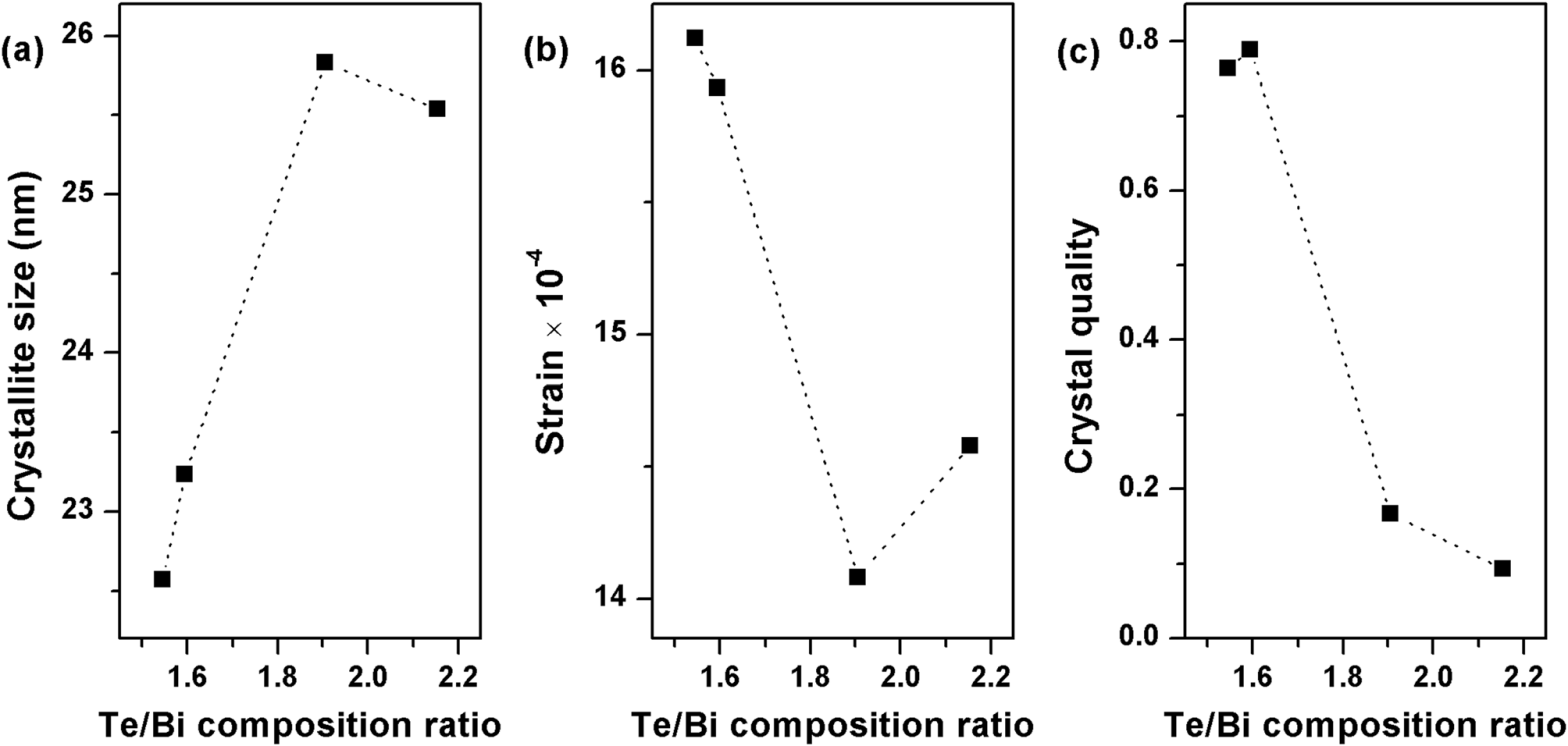
The average crystallite size, lattice strain and crystal orientation/quality of the annealed Bi–Te thin films.

Figure 8 shows the compositional dependence of the crystallographic properties of the annealed Bi–Te thin films. The average nano-crystallite size was estimated using Scherrer’s equation and presented in Fig. 8a. The crystallite size of the thin films ranged between 22 and 26 nm values and it increases when the Te/Bi ratio of the thin films was raised upto Te/Bi = 1.905. It decreased slightly for Te/Bi = 2.155 sample. The micro-strains of the thin films (Fig. 8b) decreased with Te/Bi ratio upto Te/Bi = 1.905. It slightly increased for Te/Bi = 2.155 sample. This strain behavior was consistent with the changes in thin film’s crystal structure that were observed in the XRD experiments. The film orientation changes with the gradual accumulation of Te based defects in the film structure thus relieving the internal lattice strain buildup. The crystal orientation/quality of the films (Fig. 8c) was determined from the intensity ratio of the sum of all the c-axis oriented peaks, Σ[00 l], to the sum of all of the Bi_2_Te_3_ peaks, Σ[hkl]. The highest intensity ratio, about 0.8, was measured for near stoichiometric thin film samples which indicate that these thin films have relatively high degree of c-axis orientation. The intensity ratio drastically decreases to a value < 0.2 for highly off-stoichiometric film samples. These results suggests that the introduction of Te based defects in the annealed Bi–Te thin films results in a larger nano-crystallite size, lower internal strain and lower film crystallinity. Furthermore, smaller crystallite sizes and larger internal strains were estimated for the annealed films when compared with the values that were obtained for thin films deposited at high substrate temperatures.

**Figure 9:**
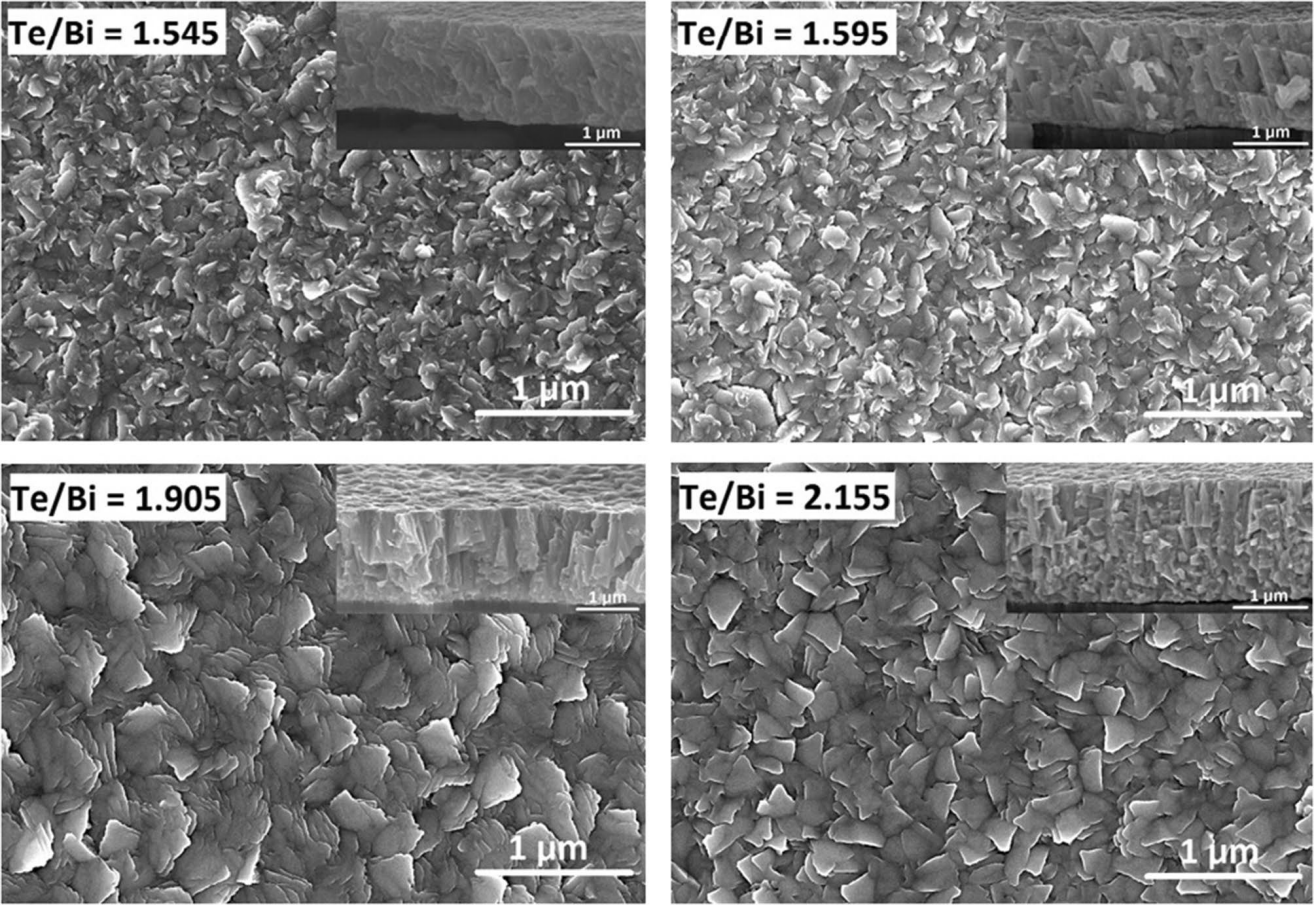
SEM images showing the surface and cross-sectional (inset) morphologies of the Bi–Te thin films after thermal annealing.

The effect of substrate temperature during deposition also had an impact on the surface and cross-sectional morphology of the resulting thin films. Figure 9 shows the SEM surface and cross-sectional images of the thin films after annealing. The grain size and shape were very different from those of the samples deposited at high substrate temperatures. The surface microstructures also showed that the grains were more compact and distributed in an ordered fashion. This could be explained by considering that the thin films were annealed at a relatively lower temperature for a considerable length of time, and at a low ramping rate of 2.5 °C/min from RT to 280 °C. This ensured that during annealing process the as-deposited film atoms had enough time to diffuse uniformly within the film volume, reach energetically favorable/stable lattice points and form a continuous, less porous and well-ordered crystallized structure. This microstructural evolution resulting from annealing is reportedly a result of the phase transformation, involving the heterogeneous nucleation and growth of new grains of stable Bi_2_Te_3_ phase from the as-deposited metastable rock salt structure^34^. As shown in the cross-sectional images, the annealed near stoichiometric thin films had a uniform thickness with well-ordered morphologies and exhibited a compact (00* l*) c-axis oriented structure. In these thin films, each oriented column was likely to be formed by a large number of thin in-plane layers stacked along the c-axis. Furthermore, significant microstructural differences were observed in the thin films with excessive deviation from the stoichiometric composition. For example, an interconnected/truncated triangular grain structure with more obvious grain boundaries was observed in the surface image of the thin film with a Te/Bi ratio of 2.155. In this case, the cross-sectional image revealed a particle like structure with occasional narrow columns, without the preferred c-axis orientation observed in the case of the stoichiometric samples. Therefore, we may infer from these results that in the Bi_2_Te_3_ system, the structural properties are unique and strongly dependent on the composition and can possibly be predicted for a specific film deposition condition.

**Figure 10:**
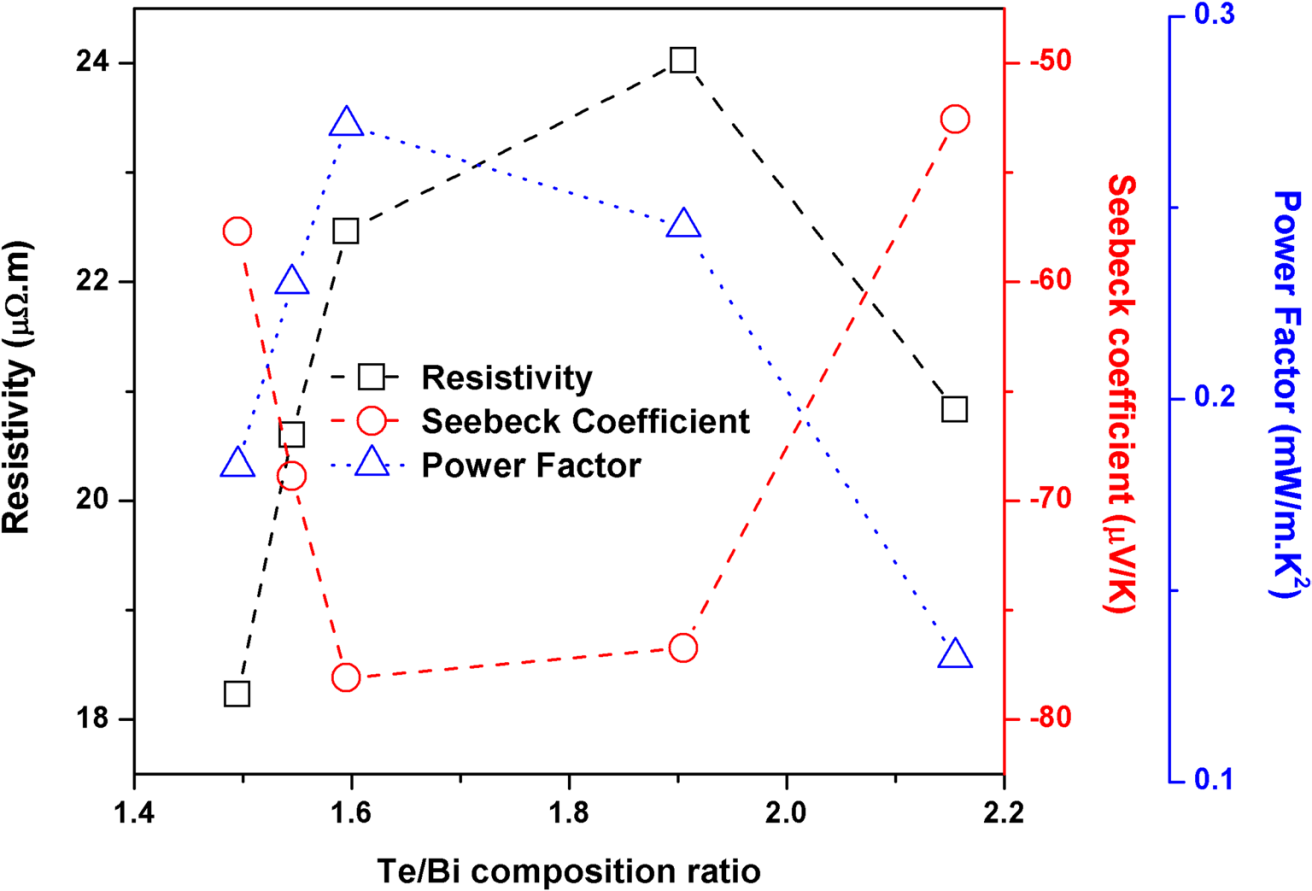
The electrical resistivity, Seebeck coefficient and PF of the annealed Bi–Te thin films.

The measured electrical resistivity of the annealed thin films increased with increasing Te/Bi ratio until Te/Bi = 1.905, as shown in Fig. 10. It then decreased once the Te content was increased further. The low electrical resistivity of the near stoichiometric thin film could be explained in terms of enhanced electron transport; this was expected due to the presence of the oriented layered structure, which reduced the grain boundary and defect interface scattering and therefore provided a preferential path for electronic conduction. On the other hand, the increase in the electrical resistivity with increasing Te/Bi ratio is considered a consequence of enhanced carrier scattering at the Bi_2_Te_3_/Te interface as well as the increased microstructural randomness and grain boundaries defects as witnessed in XRD and SEM experiments. The slight decrease of electrical resistivity for Te/Bi = 2.155 sample is then explained by a considerably high n-type doping provided by the excessive Te based defects which masked the carrier scattering effects mentioned above.

The variation in the Seebeck coefficient with Te/Bi ratio for annealed thin films is shown in Fig. 10. The Seebeck coefficient increased when the Te/Bi ratio of the thin films increased from 1.495 to 1.595. However, it decreased upon further increases in the thin film Te content. The significantly lower Seebeck coefficient observed for thin film with a Te/Bi ratio of 2.155 is a consequence of high concentration of Te based donor type defects and is consistent with the lower electrical resistivity measured for this sample. The highest value of the Seebeck coefficient, approximately − 78 µV/K, was obtained for the film with a Te/Bi ratio of 1.595. Comparable Seebeck coefficients of approximate values − 75 µV/K^23^, − 87 µV/K^24^ and − 85 µV/K^25^ were measured for sputter deposited Bi_2_Te_3_ thin films annealed at a relatively higher temperature of 300 °C. Overall, the thermoelectric power values obtained for the annealed films were considerably lower. This observation can be explained by the fact that the compact and ordered structure (with low surface roughness, porosity and defects) also had an impact on the predominant scattering mechanisms in the thin film structure. The Seebeck coefficient is understood to be directly proportional to the carrier scattering parameter^38^. The enhanced carrier motion that accompanies the well-connected ordered crystal structure implies that the carrier scattering process decreases which may explain the relatively low Seebeck coefficients that were measured for this set of prepared samples.

The PFs of the annealed thin films were higher for thin films with stoichiometric compositions (Fig. 10). The highest PF value of 0.27 mW/m K^2^ was obtained for the thin film with a Te/Bi ratio that was slightly higher than stoichiometry (Te/Bi = 1.595 sample). Due to its square dependence, the PF depends disproportionately on the value of the Seebeck coefficient. Huang et al.^23^ and Cai et al.^24^ reported comparable RT power factor values of 0.4 mW/m K^2^ and 0.27 mW/m K^2^ respectively for sputter deposited Bi_2_Te_3_ thin films annealed at 300 °C. Furthermore, for annealed samples, the maximum PF was less than half of the maximum PF obtained when deposition was carried out at high substrate temperatures. Apart from the previously discussed effect of the reduced carrier scattering on the Seebeck coefficients of the annealed thin films, this smaller value may also be partially explained by our use of a low annealing temperature, of 280 °C (as opposed to the high temperature deposition, which was carried out at a substrate temperature of 300 °C), which was selected to prevent Te re-evaporation from the thin films. We expect that an increase in the annealing temperature may be accompanied by a shortened annealing duration, which would improve the transport properties. This was demonstrated in several previous studies^23–25,29^ where Te re-evaporation from the thin films, due to annealing temperatures of 300 °C or above, was controlled/tolerated for better thermoelectric efficiency.

**Figure 11:**
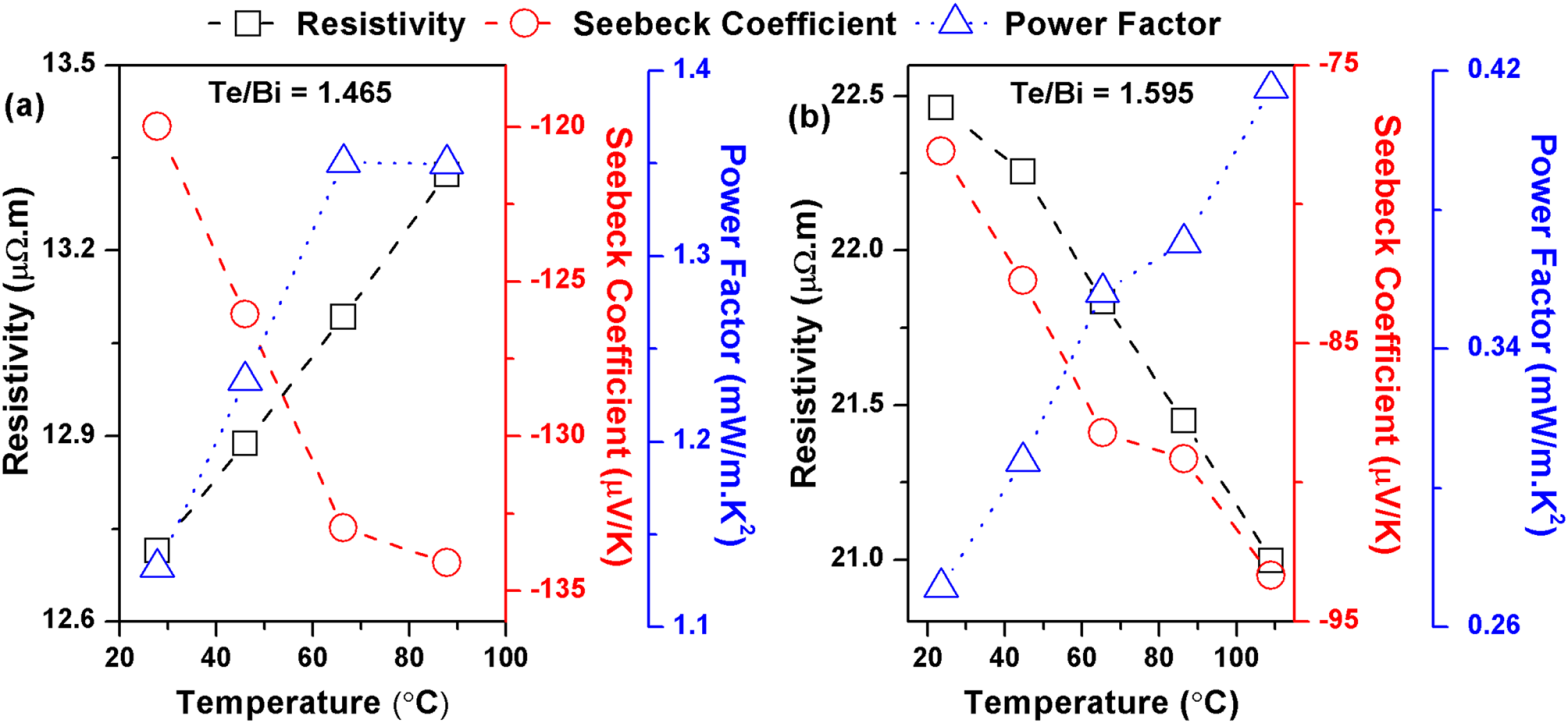
The temperature dependence of the thermoelectric properties of the best Bi–Te thin films.

The temperature dependence of the thermoelectric properties for thin films with the best measured performance in the two sets of prepared thin films is shown in Fig. 11. The PF increases with the temperature to the maximum values of 1.35 mW/m K^2^ and 0.41 mW/m K^2^ for Te/Bi = 1.465 and Te/Bi = 1.595 thin films respectively. A comparison of this study with the previous ones on Bi_2_Te_3_ thin films (since year 2011) is shown in Table 4. The maximum PF observed in this study was greater than or similar to the PFs observed in the thin films prepared by thermal evaporation^18,19,21^, sputtering^24,28–37^, PLD^39^, MBE^57^ and electrodeposition^41,42^. However, Bi_2_Te_3_ thin films with better thermoelectric properties were reported in several other studies^26,27^, indicating the need to further improve the thermoelectric efficiency of the thin film samples prepared on AlN coated Stainless Steel foil. The thermal conductivity could be favorably affected by electron/phonon scattering at the interface between Bi_2_Te_3_ and excess Te atoms. Therefore, although the PF values of off-stoichiometric films are low, their ZT values could be comparable to the stoichiometric films. It would be of great interest to measure the thermal conductivity of the thermoelectric films prepared in this study to calculate the figure of merit, ZT. However, this would require a dedicated thermal conductivity measurement setup, which we do not currently possess.

**Table 4:** Comparison of the Bi–Te thin film properties and growth methods published in the literature (since year 2011) with the present work.

References	Deposition method	Seebeck coefficient (µV/K)	Power factor (mW/m K^2^)
This work	Thermal evaporation	− 134	1.35
Ref^21^	Thermal evaporation	− 85	0.925
Ref^28^	Sputtering	− 85	1.14
Ref^34^	Sputtering	− 250	1.25
Ref^41^	Electrochemical deposition	− 58.3	0.35
Ref^27^	Sputtering	− 205	2.4
Ref^39^	Pulsed laser deposition	− 91	0.19
Ref^42^	Electrochemical deposition	− 110	1.093
Ref^26^	Sputtering	− 199	2.53
Ref^57^	MBE	− 182	0.9
Ref^24^	Sputtering	~ − 140	1.1
Ref^29^	Sputtering	− 160	0.72
Ref^18^	Thermal evaporation	− 132	0.605
Ref^37^	Sputtering	− 150	1.125
Ref^31^	Sputtering	− 193	0.97
Ref^30^	Sputtering	~ − 55	0.6
Ref^59^	Screen printing	− 138.4	0.192
Ref^35^	Sputtering	− 173	1.31
Ref^33^	Sputtering	− 92.4	0.709
Ref^32^	Sputtering	− 122	1.2
Ref^60^	Solution based synthesis	~ −150	0.89
Ref^36^	Sputtering	− 50	1.145
Ref^19^	Thermal evaporation	~ 200	1.020

## Conclusion

We grew n-type Bi–Te thermoelectric thin films on AlN-coated stainless steel foil using the vacuum thermal co-evaporation method and evaluated the thermoelectric PF to determine the optimum growth conditions. The results of our SEM, EDS and XRD experiments showed that we fabricated well crystallized thin films differing in composition, microstructure and crystal orientation, making it possible to establish the relationship between the parameters of these materials and their thermoelectric properties. Polycrystalline thin films with columnar micro-structure and multi-oriented crystal structure were obtained in the case of the thin films deposited at high substrate temperatures, whereas the annealed stoichiometric thin films have a unique ordered microstructure consisting of in-plane layers preferentially stacked along the (00* l*) c-axis orientation. In the latter case, the preferred orientation was found to depend on the film composition. The thermoelectric properties were also found to be hugely dependent on the deposition temperature and film composition. We observed that the best thin film properties were obtained in the case of nearly stoichiometric samples, and the highest thermoelectric PFs were generally obtained for thin films with the highest Seebeck coefficients. For the best thin film synthesized at a substrate temperature of 300 °C, the maximum RT Seebeck coefficient and PF values were − 120 µV/K and 1.13 mW/m K^2^, which were observed in the thin film with Te/Bi = 1.465. In the case of thin films deposited at RT then annealed at 280 °C, the maximum RT Seebeck coefficient and PF were − 78 µV/K and 0.27 mW/m K^2^, respectively. These values were obtained for the thin film with Te/Bi = 1.595. We expect that increasing the annealing temperature will improve the thermoelectric properties of these films. The PFs for the best reported films in each discussed set rises to the values of 1.35 mW/m K^2^ and 0.41 mW/m K^2^ respectively when measured as a function of temperature. The excess Te atoms in the off-stoichiometric thin films are observed to act as both electron donor and electron blocker and therefore significantly influence the electrical (and thermal) properties. These experimental data demonstrate that efficient thermoelectric thin films with controlled microstructures and crystal orientations can be prepared and characterized on AlN-coated stainless steel foil. The thin film technology employed in this work provides a feasible route for fabricating a flexible cross-plane thermoelectric micro-generator, provided that the foil thickness is reduced to approximately 100–150 µm.
